# Synaptopathy: presynaptic convergence in frontotemporal dementia and amyotrophic lateral sclerosis

**DOI:** 10.1093/brain/awae074

**Published:** 2024-03-07

**Authors:** Emma L Clayton, Laura Huggon, Michael A Cousin, Sarah Mizielinska

**Affiliations:** UK Dementia Research Institute at King’s College London, London SE5 9RT, UK; Department of Basic and Clinical Neuroscience, Institute of Psychiatry, Psychology and Neuroscience, King’s College London, Maurice Wohl Clinical Neuroscience Institute, London SE5 9RT, UK; UK Dementia Research Institute at King’s College London, London SE5 9RT, UK; Department of Basic and Clinical Neuroscience, Institute of Psychiatry, Psychology and Neuroscience, King’s College London, Maurice Wohl Clinical Neuroscience Institute, London SE5 9RT, UK; Centre for Discovery Brain Sciences, University of Edinburgh, Edinburgh EH8 9XD, UK; Muir Maxwell Epilepsy Centre, University of Edinburgh, Edinburgh EH8 9XD, UK; Simons Initiative for the Developing Brain, University of Edinburgh, Edinburgh EH8 9XD, UK; UK Dementia Research Institute at King’s College London, London SE5 9RT, UK; Department of Basic and Clinical Neuroscience, Institute of Psychiatry, Psychology and Neuroscience, King’s College London, Maurice Wohl Clinical Neuroscience Institute, London SE5 9RT, UK

**Keywords:** frontotemporal dementia, amyotrophic lateral sclerosis, synaptopathy, presynaptic, synaptic vesicle, synaptic dysfunction

## Abstract

Frontotemporal dementia and amyotrophic lateral sclerosis are common forms of neurodegenerative disease that share overlapping genetics and pathologies. Crucially, no significantly disease-modifying treatments are available for either disease. Identifying the earliest changes that initiate neuronal dysfunction is important for designing effective intervention therapeutics. The genes mutated in genetic forms of frontotemporal dementia and amyotrophic lateral sclerosis have diverse cellular functions, and multiple disease mechanisms have been proposed for both. Identification of a convergent disease mechanism in frontotemporal dementia and amyotrophic lateral sclerosis would focus research for a targetable pathway, which could potentially effectively treat all forms of frontotemporal dementia and amyotrophic lateral sclerosis (both familial and sporadic).

Synaptopathies are diseases resulting from physiological dysfunction of synapses, and define the earliest stages in multiple neuronal diseases, with synapse loss a key feature in dementia. At the presynapse, the process of synaptic vesicle recruitment, fusion and recycling is necessary for activity-dependent neurotransmitter release. The unique distal location of the presynaptic terminal means the tight spatio-temporal control of presynaptic homeostasis is dependent on efficient local protein translation and degradation.

Recently, numerous publications have shown that mutations associated with frontotemporal dementia and amyotrophic lateral sclerosis present with synaptopathy characterized by presynaptic dysfunction. This review will describe the complex local signalling and membrane trafficking events that occur at the presynapse to facilitate neurotransmission and will summarize recent publications linking frontotemporal dementia/amyotrophic lateral sclerosis genetic mutations to presynaptic function. This evidence indicates that presynaptic synaptopathy is an early and convergent event in frontotemporal dementia and amyotrophic lateral sclerosis and illustrates the need for further research in this area, to identify potential therapeutic targets with the ability to impact this convergent pathomechanism.

## Teaser

Frontotemporal dementia and amyotrophic lateral sclerosis share overlapping genetics and pathologies. Clayton *et al.* summarise recent evidence suggesting that presynaptic dysfunction is an early and common event in both diseases, and could represent a target for future disease-modifying therapies.

## Frontotemporal dementia and amyotrophic lateral sclerosis

Frontotemporal dementia (FTD) and amyotrophic lateral sclerosis (ALS) are two devastating neurodegenerative diseases that, despite significantly different clinical presentations, are intrinsically linked and now considered to be on a disease continuum. FTD is the second most common form of young onset dementia,^1^ and conversely to the classic symptoms of dementia-associated memory loss, presents primarily with changes to behaviour and language.^2^ These changes are directly associated with atrophy of frontal and temporal lobes, respectively. Reported incidence rates for FTD range from 0.0–0.3 per 1000 person-years.^3^ There are several subtypes of FTD, grouped by different clinical symptoms, with the behavioural variant (bvFTD) most commonly overlapping with ALS. At the other end of the continuum, ALS is the most common motor neuron disease, with a global incidence of 1.9 per 100 000.^4^ Both upper motor neurons in the motor cortex and lower motor neurons in the spinal cord degenerate in ALS, resulting in progressive muscle weakness and early death, most commonly due to respiratory compromise within 5 years.^5^ It is considered a clinical spectrum because people initially diagnosed with either FTD or ALS can subsequently develop the opposing disorder; specifically, between 4–9% of people diagnosed with FTD develop ALS,^6,7^ and 50% of people with ALS show cognitive impairment.^8^ However, the continuum does not end here; these disorders also show remarkable overlap at both the genetic and pathological level.

### Genetics

**Figure 1:**
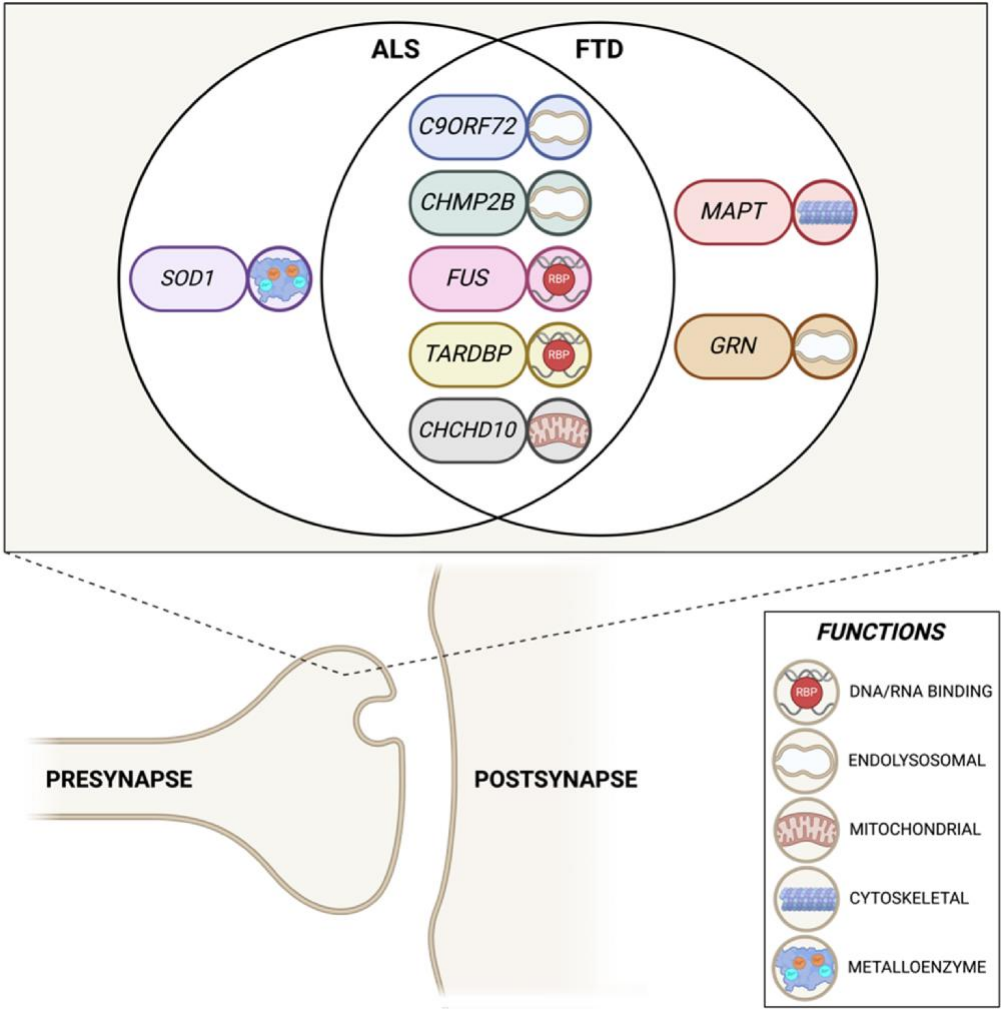
**Major known genes causative for ALS and FTD associated with synaptic dysfunction**. The genetics of the two neurodegenerative disorders overlap significantly, with many genes found to be implicated in the pathology of both amyotrophic lateral sclerosis (ALS) and frontotemporal dementia (FTD). Emerging evidence suggests that misregulation of these genes contribute to synaptic dysfunction and loss in disease. The function of each of the encoded proteins is also shown. Created with BioRender.com.

FTD and ALS both have heritable components, with 40% of FTD being considered familial and 10% of ALS.^9,10^ Over the last three decades, significant advances have been made in the identification of the genetic mutations which comprise this heritability, although in both, more than a third remain unknown. At one end of the spectrum, mutations in *PGRN* (encoding the secretory protein progranulin) and *MAPT* (encoding the microtubule binding protein tau) almost only ever cause FTD, whereas mutations in the antioxidant enzyme *SOD1* only cause ALS.^11^ However, the majority of genetic mutations can lead to both disorders. By far the most frequent of these, in European-associated populations, is the *C9ORF72* repeat expansion mutation, discovered in 2011.^12,13^ In general, mutations cluster into pathways associated with RNA homeostasis (*TARDBP*, *FUS*, *ATXN2*, *HNRNPA1/A2B1*, *MATR3*), autophagy (*TBK1*, *OPTN1*, *SQSTM1*, *UBQLN2*, *VCP*, *CHMP2B*, *VAPB*, *ALS2* and *C9ORF72*) and less frequently with cytoskeletal dynamics (*PFN1*, *DCTN1*, *NEFH*, *TUB4A* and also *MAPT* in FTD), mitochondria (*CHCHD10* and *SOD1* in ALS) and DNA damage (*FUS*, *NEK1*, *C21ORF2*, *SPG11*).^14^ Importantly for this review, many of these genetic forms of FTD/ALS are also associated with synaptopathy (Fig. 1).

### Pathology

In line with most neurodegenerative disorders, post-mortem central nervous tissue of FTD and ALS cases both exhibit gliosis and aggregated protein deposits.^15^ These protein aggregates are frequently ubiquitinated and labelled with the autophagy receptor p62 (encoded by *SQSTM1*), as part of the cell’s attempt to target the misfolded proteins for degradation.^16^ However, the main protein constituents of the deposits vary and are used to classify cases into pathological subtypes. In FTD, these form subtypes of frontotemporal lobar degeneration (FTLD) pathology: ∼40% of cases exhibit inclusions primarily composed of TDP-43 (encoded by *TARDBP*, FTLD-TDP), ∼40% with tau pathology (FTLD-tau), less frequently FTLD-FUS/FET with inclusions of the FET proteins FUS, EWS and TAF15, and finally FTLD-U of unknown composition.^17^ In ALS, the vast majority (∼97%) of cases exhibit TDP-43 pathology, with the remaining cases exhibiting SOD1 or FUS inclusions, all found both in lower and upper motor neurons.^18^

Both the genetics and pathology of FTD and ALS are interlinked, but not in a linear manner. For example, mutations in *TARDBP* are associated with TDP-43 pathology in both FTD and ALS, but mutations in *FUS* are only linked with FUS pathology in ALS. FTLD-FUS/FET pathology in FTD differs in that it is composed of hypomethylated FUS with EWS and TAF15 but not transportin-1, as in ALS.^19^ While FTD-causative *FUS* mutations are very rare,^20,21^ FUS pathology has been reported in a FTD patient with *FUS* mutation.^22^ At the ends of the spectrum, SOD1 mutations occur with SOD1 aggregates in ALS^23^ and tau mutations with FTLD-tau.^24^ Finally, despite differing functions and clinical associations, both *PGRN* and *C9ORF72* mutations exhibit TDP-43 pathology. A notable exception to these groupings is *CHMP2B* mutations, which are classified as FTLD-U.^25^

### Disease pathomechanisms

The cellular pathways that have been linked with FTD and ALS are many and diverse,^26-29^ which may be expected considering the range in clinical presentation, genetics and pathology highlighted above. However, there are significant areas of convergence. These may arise due to the primary role of the associated genetic mutations including, as mentioned above, most frequently RNA homeostasis and autophagy, but also to cytoskeletal dynamics, mitochondrial function and DNA damage.^30-32^ Other key mechanisms come from the characterization of cell or animal models of genetic disease, including dysfunction in nucleocytoplasmic shuttling,^33^ transport along axons and dendrites,^34,35^ response or resistance to stress: stress granule dynamics, oxidative stress, glutamate excitotoxicity,^36-38^ and synaptic physiology—the latter of which will be discussed in more detail in this review. Finally, although neurons are the focus of most mechanistic studies in ALS and FTD, gliosis is a dominant feature in post-mortem tissue and disease models and, therefore, likely plays a crucial or modulatory role.^15,39^ Indeed, neuroglia have roles in the modulation of synaptic activity and maintenance, which where relevant to the presynapse, will also be discussed.

## The presynaptic terminal

By definition, the locus of dysfunction in synaptopathic disease is the synapse. Extensive research has revealed general synaptic disease signatures that may culminate in either or both FTD and ALS, which predominantly involve postsynaptic studies.^40-42^ In contrast, research into presynaptic disease mechanisms is considerably less advanced. The most likely explanations for this are the relative accessibility of the postsynapse to electrophysiological analysis and the prevailing dogma that dysfunction in neurotransmitter release would be either incompatible with life or, at a minimum, would present in early life. This latter scenario has been confirmed for a series of neurodevelopmental disorders^43,44^; however, there is emerging evidence that presynaptic dysfunction also contributes towards neurodegenerative conditions.

**Figure 2:**
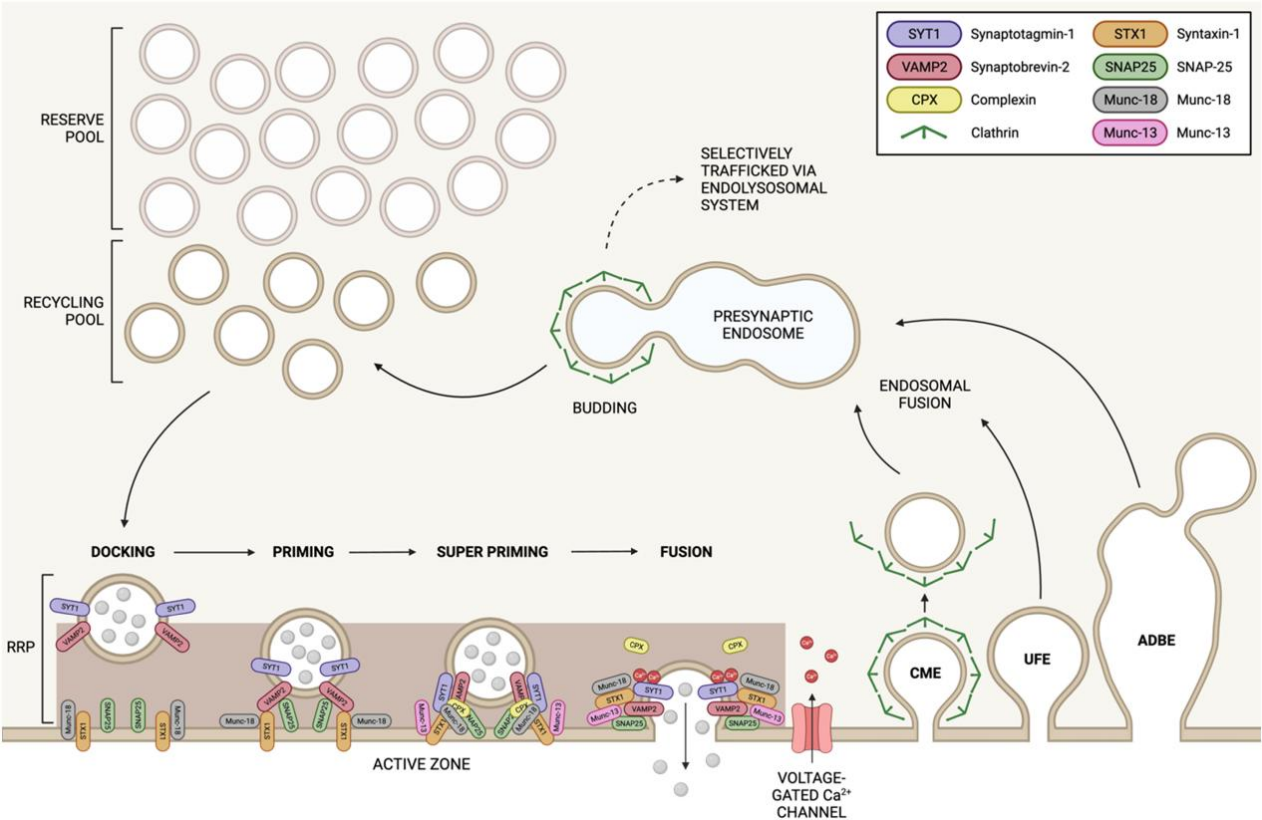
**Synaptic vesicle trafficking in the pre-synaptic terminal**. Schematic representation showing synaptic vesicle exocytosis, endocytosis and recycling. Synaptic vesicles from the recycling pool undergo docking at the presynaptic active zone to form the readily releasable pool (RRP). The docked synaptic vesicles are primed for fusion through interactions between synaptobrevin-2 (VAMP2) and the plasma membrane proteins SNAP-25 and syntaxin-1 (STX1) to form the SNARE complex. Munc-18 and Munc-13 also support the complex assembly. Super priming results from the association of the calcium-binding protein, synaptotagmin-1 (SYT1), and complexin (CPX) with the SNARE proteins to stabilize the complex further. An arriving action potential then triggers the entry of Ca^2+^ via voltage-gated channels. Ca^2+^ binds to SYT1, leading to vesicle fusion with the membrane and neurotransmitter release. Vesicles are retrieved from the plasma membrane via clathrin-mediated endocytosis (CME), ultrafast endocytosis (UFE) or activity-dependent bulk endocytosis (ADBE). The latter two pathways generate endosomes directly from the plasma membrane, with CME initiated at this site, but completing on these presynaptic endosomes. Synaptic vesicles are reformed by clathrin coats budding from endosomes or vesicular carriers selectively trafficked via the endolysosomal system for degradation. Created with BioRender.com.

The presynaptic terminal is populated by neurotransmitter-containing synaptic vesicles. It is the activity-dependent fusion (exocytosis) of these synaptic vesicles that mediate neurotransmitter release and thus modulate postsynaptic function (Fig. 2). There are ∼200–300 synaptic vesicles at a typical small central presynapse, however, approximately only half are able to be mobilized by neuronal activity.^45,46^ This small pool of synaptic vesicles, termed the recycling pool (RP), which has a subset of synaptic vesicles immediately available for fusion, the readily releasable pool (RRP), has to be replenished rapidly and efficiently to sustain neurotransmission. This is done via a series of synaptic vesicle endocytosis modes that operate with different timescales and are triggered by different activity patterns.^47^

### Synaptic vesicle fusion and neurotransmitter release

Neurotransmitter release is triggered by the invasion of an action potential into the presynapse, which opens voltage-gated calcium channels, resulting in the fusion of synaptic vesicles to the presynaptic membrane and release of contents into the synaptic cleft.^48,49^ This release is highly synchronous and is mediated by the calcium-sensing synaptic vesicle protein synaptotagmin-1.^50^ However, for exocytosis to occur, the synaptic vesicle must first proceed through a series of defined molecular events. The first stage is the physical attachment of the synaptic vesicle to the active zone in a process called ‘docking’. The active zone is a defined subcellular structure at the presynaptic plasma membrane that coordinates and organizes specific molecules to optimize synaptic vesicle fusion.^51^ The docked synaptic vesicle is then made competent for release in a series of molecular steps termed ‘priming’.^49,52^ Priming involves the coordinated assembly of the SNARE (soluble *N*-ethylmaleimide sensitive factor attachment protein receptor) complex, which is the minimal machinery required to fuse a synaptic vesicle.^53^ The SNARE proteins are synaptobrevin-2 (an integral synaptic vesicle protein), syntaxin-1 (an integral plasma membrane protein) and SNAP-25 (a peripheral plasma membrane protein).^54,55^ Synaptic vesicle fusion is driven by the progressive association of coiled-coil domains called SNARE motifs on each protein. These bring the synaptic vesicle and plasma membranes into close opposition until it is thermodynamically favourable for the membranes to fuse.^53^ However, membrane fusion mediated by the SNARE complex alone is extremely slow, therefore priming ensures that its assembly is optimal and synchronous with neuronal activity.^49,52^ Key players in the priming process are Munc-13, Munc-18 and complexin, which work in concert to chaperone individual SNARE proteins in a sequential series of interactions. Munc-18 positions syntaxin-1 in its closed conformation (which is inaccessible to other SNARE motifs) and synaptobrevin-2 in close proximity due to multivalent interactions.^56,57^ Munc-13 then opens up syntaxin-1, while simultaneously orienting both synaptobrevin-2 and SNAP-25 SNARE motifs in the correct configuration for optimal complex assembly.^58^ This results in formation of a SNARE complex that is resistant to the action of the ATPase NSF (*N*-ethylmaleimide sensitive factor), which disassembles SNARE complexes (see later). SNARE-dependent synaptic vesicle fusion is prevented at this stage by complexin, a small cytoplasmic protein containing a coiled-coil domain that intercalates into the SNARE complex, preventing the final stages of assembly.^59^ This stabilizes the primed SNARE complex in a conformation that is sometimes referred to as ‘super-primed’.^49^ Super-priming may also be a result of the simultaneous association of complexin with synaptotagmin-1 and the SNARE complex.^60^ Upon calcium influx, synaptotagmin-1 binds calcium and displaces complexin from the SNARE complex, while simultaneously interacting with plasma membrane lipids^61,62^ (and potentially the SNARE complex^60^) to couple activity to synaptic vesicle fusion and thus neurotransmitter release. After exocytosis has occurred, the SNARE complex (which is now in the plasma membrane) is disassembled to free syntaxin-1 and SNAP-25 to enter new SNARE complexes and allow synaptobrevin-2 to be trafficked back to new synaptic vesicles. As noted above, this function is performed by the ATPase NSF, which is recruited to the SNARE complex by the small molecule a-SNAP (soluble NSF-attachment protein).^49,52^

### Synaptic vesicle endocytosis

As described, the number of synaptic vesicles within the RP is relatively small, making the accurate and rapid reformation of synaptic vesicles after their fusion essential to maintain neuronal communication. This is mediated by a series of different endocytosis modes and mechanisms.^47^ The dominant modes of membrane retrieval at the presynapse are fast, actin-driven, fluid phase uptake pathways such as ultrafast endocytosis (UFE) and activity-dependent bulk endocytosis (ADBE).^63-65^ Both UFE and ADBE form large invaginations at the presynaptic plasma membrane, which fission to form endosomes.^63,64^ UFE and ADBE are currently recognized as independent endocytosis modes as they appear to be triggered by either mild or strong patterns of activity, respectively.^63,64^ However, they share very similar molecular mechanisms.^63,65-70^ Therefore, this discrimination may soon become redundant, with the result that one unifying endosomal pathway retrieves synaptic vesicle membrane during neuronal activity.

The presynaptic endosomes formed via either UFE or ADBE then generate synaptic vesicles to repopulate the recycling pool.^71-73^ Importantly this process is dependent on the action of both clathrin and its adaptor protein complexes.^67,73,74^ This machinery recognizes synaptic vesicle cargo via specific sorting motifs, allowing synaptic vesicles to be generated that have the required proteins in the correct stoichiometry.^75^ The process of clathrin-mediated endocytosis (CME) was previously assumed to occur at the plasma membrane, however, these events appear to be absent at physiological temperatures.^67,73^ This suggested that while CME initiates at the plasma membrane, it completes on either large UFE/ADBE invaginations or presynaptic endosomes. This is a result of the speed of both UFE and ADBE in generating endosomes, and the relatively slow formation and invagination of clathrin-coated pits. Intriguingly, not all synaptic vesicle cargo is sorted back to newly generated synaptic vesicles from these endosomes, with some selectively trafficked via the endolysosomal system.^76^ Therefore, these presynaptic endosomes can be envisaged as sorting stations that can adjust the composition of synaptic vesicles to the demands placed on the presynapse by neuronal activity.^77^ Conversely, dysfunction in the endolysosomal system may result in synaptic vesicles with an altered inventory of molecules that could impact the probability of neurotransmitter release.^77^

## Evidence for presynaptic synaptopathy in FTD/ALS

### Frontotemporal dementia

#### Tau

Numerous mutations in the *MAPT* gene, which encodes the protein Tau, have been identified in FTD.^78,79^ Tau is a microtubule binding protein that supports axonal transport and contributes to stability of microtubules.^80,81^ Six isoforms of tau are produced with differential inclusion of two N-terminal domains (termed 0N, 1N or 2N) and addition of three or four microtubule binding domains (3R or 4R). During development, 3R tau is predominant, with 4R isoforms increasing in early life and reaching equal levels with 3R in adulthood.^82^ All isoforms are found in the human CNS, but are believed to have different physiological functions. In disease conditions, tau becomes hyperphosphorylated, dissociates from microtubules and deposits in aggregates.^83^ FTD causative mutations occur throughout the *MAPT* gene but are enriched in the C-terminal region in and around the microtubule binding domains; indeed some are specific to the 4R isoforms as they are encoded in exon 10.^84^ Mutations frequently alter tau splicing^85^ and decrease the ability of tau to interact with microtubules, thereby increasing cytosolic tau aggregation.^86^

It is important to note that in addition to FTD/ALS, tau pathology is extensively studied in the context of several other neurodegenerative conditions such as Alzheimer’s disease (AD), progressive supranuclear palsy and corticobasal syndrome. These diseases are collectively known as tauopathies, where the formation of tau neurofibrillary tangles is a hallmark pathology of disease. The isoform composition of these intracellular filaments (3R, 4R, 3R + 4R) allows the pathological grouping of these diseases.^83^ Whether tau exerts a physiological or pathological influence at the synapse has been extensively discussed (for a comprehensive review, see Robbins *et al.*^87^). It is also important to mention non-cell autonomous considerations when modelling tau physiology or pathology. For example, microglia play an active role in targeting synapses with accumulated tau and can modulate synaptic tau spread (for a review, see Vogels *et al.*^88^). In the context of synaptopathy in FTD/ALS, we will summarize the evidence for a presynaptic physiological role of tau and a corresponding pathological contribution to synaptopathy.

##### Synaptic activity and tau spread

Numerous studies have now shown that both synaptic activity and synaptic connectivity are important in tau spread, showing that functional synaptic units are a focal point for pathogenic tau propagation.

Stimulating neuronal activity induces tau release from healthy, mature cortical neurons.^89^ Interestingly, the pattern of spread of tau propagation (with rapid and progressive neurofibrillary tangle pathology) is determined by connectivity, not proximity,^90^ indicating a synaptic component to tau spreading. Indeed, the physical presence of synaptic contacts between neurons facilitates tau pathology propagation.^91^ In elegant experiments designed to explore this phenomenon further, neurons derived from a transgenic mouse line expressing human tau (hTau) with the P301L mutation (rTg4510, ‘donor’) were cultured with neurons from a tau knockout-GFP line (‘recipient’), which showed that endogenously generated hTau can transfer from cell to cell via the extracellular medium.^92^ Furthermore, the authors found that tau release and cell-to-cell transfer are enhanced by stimulating neuronal activity.^92^

Whilst this evidence made it clear that tau is spread from cell to cell via synaptic contacts, at the time, it was unknown whether tau accumulated as a cause or consequence of synapse loss. Innovative work has addressed this issue, using a mouse model again expressing the human P301L mutant tau, in addition to fluorescent markers labelling both the cytoplasm (tdTomato) and presynaptic terminals (synaptophysin-EGFP). In this study, the neuropsin promoter drove tTA expression, restricting transgene expression primarily to the entorhinal cortex. This clever fluorescent tagging approach allowed visualization of the synaptic terminals of human tau-expressing neurons. Using this model, the authors demonstrated that the propagation of tau through anatomically connected brain regions was not due to the degeneration of presynaptic terminals and was, in fact, an early feature of the disease process.^93^

Synaptic activity as a driver of tau spread has also been investigated in the oligosynaptic giant fibre system in the adult *Drosophila* CNS, where normal or mutant human tau isoforms affect distinct synaptic parameters. For example, the 0N3R isoform increased failure rate upon high-frequency stimulation with a significantly increased refractory period, whilst 0N4R compromised stimulus conduction and response speed at a specific cholinergic synapse in an age-dependent manner. Expressing the R406W mutant of 0N4R induced mild, age-dependent conduction velocity defects.^94^ This work is important in the context of human tau isoform expression, as the dominant forms of tau isoform alter as humans age. As stated earlier, 3R tau predominates in development, whereas levels of 4R tau increase during early life, with 4R tau levels matching 3R in adulthood.^82,95^ However, the developmental regulation of tau splicing is known to be dysregulated in neurons derived from FTD patients with the 10 + 16 mutation in MAPT,^96^ a splice site mutation that destabilizes tau RNA and leads to a 2–6-fold increase in the inclusion of exon 10, the exon responsible for production of the 4R isoform^97^

Further studies investigating the role of neuronal activity in the pathophysiology of tau spread used adenoviruses to express hTau and an excitatory DREADD (designer receptors exclusively activated by a designer drug) in the ventral hippocampus of wild-type mice, P301L hTau-expressing mice, or tau knockout mice. DREADD-mediated stimulation of neuronal activity increased hTau pathology and spread to distal brain regions irrespective of mouse model compared to unstimulated controls.^98^

While the above evidence highlights that increased neuronal activity enhances tau release and propagation, it is important to note that an alternative study demonstrated that synaptic activity can protect against AD and FTD-like pathology. Chronic deep brain stimulation in 3xTg-AD transgenic mice (which contain the MAPT P301L mutation in addition to mutations in APP and PSEN1) resulted in reduced levels of tau oligomers and increased levels of synaptophysin compared to non-stimulated mice, as quantified by confocal immunofluorescence in the hippocampal CA1 region. Conversely, chronic synaptic inhibition by unilateral deafferentation increased levels of tau oligomers and reduced levels of synaptophysin.^99^ Together, these studies suggest either activity or neuron-specific vulnerability is key to tau and synaptic pathologies.

Indeed, specificity in the type of neuron vulnerable to tau pathophysiology has been identified again using the rTg4510 tauopathy mouse model expressing human P301L mutant tau. PET imaging showed that inhibitory synapses were significantly dysregulated in this model before brain atrophy at 2 months of age, while excitatory synapses stayed relatively intact at this stage. Further studies in AD have supported this concept of regional vulnerability and have identified axon plasticity genes as a link to tau in the vulnerable neuron population.^100,101^ These studies highlight critical early axo-synaptic pathophysiology and selective vulnerability of neuronal populations to tau pathophysiology.^102^

##### Synaptic localization of tau

Interestingly, in a study examining the distribution of tau protein in a different transgenic mouse model expressing human P301L tau (JNPL3) and non-transgenic littermates, human P301L tau was preferentially distributed in synaptosomes (isolated synaptic terminals), while mouse endogenous tau was more distributed in the cytosolic fraction.^103^ This work indicates species-specific physiology and the importance of using human tau to investigate disease pathomechanisms. Similarly, tau was also abundant in synaptosomes from human post-mortem samples.^104^ In the same study, it was demonstrated that potassium-induced depolarization induces the release of tau and tau fragments from synaptosomes, with increased release from AD compared to control samples.^104^ In another study using immunofluorescence imaging of synapses from AD post-mortem tissue, tau protein was detected in ∼40% of presynaptic and 50% of postsynaptic terminals, with ∼30% of hyperphosphorylated tau (PHF1) and 20% of misfolded tau in both.^105^ Using antibodies against another phospho-tau epitope (AT8) and the presynaptic marker protein synapsin, brain array tomography in AD patient brain also illustrated the presence of pathological tau at the presynapse.^106^

These works show that tau plays a physiological role at the presynaptic terminal, and post-translationally modified pathological tau is found in patient synapses in greater abundance. Delineating the physiological function of tau, and the consequence of this presynaptic burden of pathological tau, is important to our understanding of tauopathy.

##### Presynaptic function of tau

In addition to the noted localization of tau to the presynapse, numerous studies have demonstrated that tau has a function in the organization and trafficking of synaptic vesicles, which is significantly dysregulated by pathogenic variants of tau.

In a glutamatergic sensory-motor neuron (MN) synapse derived from *Aplysia ganglia*, the expression of mutant hTau (containing both P301S and K257T) in the presynaptic neurons induced synaptic weakening correlated with a reduced releasable presynaptic synaptic vesicle pool.^107^ In *Drosophila*, tau localized at presynaptic terminals and bound to synaptic vesicles via its N-terminal domain.^106^ Expression of R406W, V337M or P301L mutants of tau in the *Drosophila* model reduced synaptic transmission, synaptic vesicle recycling and fusion during sustained high-frequency stimulation. Additionally, tau mutants increased F-actin levels and reduced synaptic vesicle mobility at presynaptic terminals, increasing clustering.^106^ Expanding on the synaptic vesicle-associated role for tau, the integral synaptic vesicle protein synaptogyrin-3 was identified as a binding partner of tau on synaptic vesicles.^108^ In both fly and mouse models of tauopathy (P301L/S), the reduction of synaptogyrin-3 prevented the association of presynaptic tau with synaptic vesicles, alleviating tau-induced defects in synaptic vesicle mobility, and restoring neurotransmitter release.^108^ Investigating this further, heterozygous knockout of synaptogyrin-3 was sufficient to preserve synaptic plasticity and prevent loss of synapses in P301S mutant tau mice.^109^ Taken together, these works identify a synaptic vesicle-specific role for tau, which may contribute to early synaptic dysfunction in neurodegenerative diseases associated with tauopathy.

To define the interactome of tau in human induced pluripotent stem cell (iPSC)-derived glutamatergic neurons, Tracy *et al.*^110^ combined engineered ascorbic acid peroxidase (APEX) proximity-dependent mapping with mass spectrometry. The authors modified iPSC-derived neurons by integrating a transgene for doxycycline-inducible expression of human tau tagged with a flag epitope and APEX2. Analyses of the distinct N- and C-terminal tau-associated proteins revealed networks involved in synaptic vesicle regulation; N-terminal tau associated proteins are mostly involved in active zone docking, while C-terminal interacting proteins, such as syntaxin 1A/1B (STX1A/1B), RAB3A, RIMS1 and Mint1 (APBA1), are important for the fusion of synaptic vesicles.^110^ This important work indicates domain-specific interactions of tau with synaptic vesicle-associated proteins, implicating it as a potentially integral component of the synaptic vesicle life cycle.

The above studies, using multiple different models, highlight that numerous variables govern the role of tau at the presynaptic terminal. Thus, tau species, whether in terms of isoform, genetic mutations or post-translational modification, are extremely important to control when inferring disease-relevant mechanisms of action.

#### Progranulin

Heterozygous mutations in *GRN*, the gene that encodes progranulin, are causative for FTD and are largely nonsense or frameshift mutations that lead to progranulin loss-of-function.^111^ Progranulin, which is primarily expressed in neurons and microglia, plays a role in inflammation, with progranulin-deficient mice displaying activation of microglia and astrocytes.^112^ In addition to the role of progranulin as a secreted signalling protein, it plays a role in lysosomal degradative processes via the regulation of cathepsin and glucocerebrosidase^113^ and mouse models of PGRN deficiency present with accumulation of lysosomal proteins.^114-116^ Interestingly, complete deficiency of GRN in humans causes neuronal ceroid lipofuscinosis, a lysosomal storage disorder.^117^

As a protein dense hub of spatiotemporally controlled signalling events, synapses are also dependent on local protein degradation pathways.^118^ Thus, deficits to protein degradation pathways, such as the endolysosomal system, could mediate local imbalances to synaptic homeostasis. The first reports of a physiological dysfunction of synapses in PGRN models came from knockdown of the protein in rat primary hippocampal neurons, where the number of synaptic vesicles per synapse and the frequency of miniature excitatory postsynaptic currents were increased.^119^ In agreement, the authors also examined post-mortem brain tissue from FTD patients with PGRN haploinsufficiency and saw an increased number of synaptic vesicles per synapse.^119^ Altered synaptic connectivity and plasticity have also been reported in progranulin-deficient mice.^116^ Interestingly, Petoukhov *et al.*^120^ reported that, similarly to tau, PGRN is secreted in an activity-dependent manner from synaptic sites. The mechanism of release for PGRN shares characteristics with other large dense core vesicle cargos such as brain-derived neurotrophic factor. This may have implications for presynaptic function since application of exogenous PGRN decreases synaptic vesicle numbers,^120^ potentially limiting neurotransmission.

As PGRN is strongly expressed by microglia, there is also a clear non-cell autonomous component to PGRN-associated synaptopathy. Important work from Lui *et al.*^121^ shows that microglia-mediated synaptic pruning is a driver, rather than a consequence, of neurodegeneration caused by progranulin deficiency. During ageing, progranulin-deficient mice showed profound microglia infiltration and targeted elimination of inhibitory synapses in the ventral thalamus, which led to hyperexcitability in thalamocortical circuits and obsessive grooming. They also showed that preventing complement activation mitigated neurodegeneration and significantly reduced synaptic pruning by progranulin-deficient microglia.

Thus together, evidence shows that progranulin can play a role in presynaptic pathology via cell autonomous mechanisms involving synaptic vesicles and non-cell autonomous synaptic clearance in PGRN-FTD.

#### CHMP2B

An autosomal dominant mutation in the endosomal sorting complex required for transport (ESCRT)-III subunit CHMP2B was found to be causative for FTD in a large Danish cohort,^122,123^ and has since also been found in other families.^124^ The ESCRT complex is a membrane remodelling complex responsible for multiple membrane trafficking processes, such as membrane sealing, cytokinesis and protein degradation.^125^ In common with PGRN, neuronal lysosomal storage pathology was observed in both a mouse model of CHMP2B FTD and in patient tissue,^126^ indicating that mutations in *CHMP2B* also impair endolysosomal trafficking.

Synaptopathy in CHMP2B FTD was first reported in *Drosophila* motor neurons expressing FTD-causative CHMP2B^Intron5^, which show significant aberrant synaptic overgrowth at the neuromuscular junction (NMJ).^127^ Synaptopathy has also been reported in mouse models of CHMP2B FTD. Defects to synaptic vesicle cycling and synaptic vesicle pools and an increased number of presynaptic endosomes were found in primary cortical cultures from CHMP2B^Intron5^ mutant mice.^128^ Additionally, selective retention of synaptic vesicle-associated proteins in aged mice were observed despite significant synaptic loss.^128^ The ESCRT complex has been identified as a key driver of activity-dependent protein degradation of select synaptic vesicle proteins.^129^ Thus, the selective retention of synaptic vesicle proteins in this CHMP2B mouse model could be associated with the reported neuronal endolysosomal dysfunction. Indeed, an accumulation of endolysosomal proteins is reported in aged mice^126^ and defective neuronal endolysosomal trafficking in primary cortical cultures from this transgenic mouse model.^130^

This synaptopathy has been recapitulated in a further CHMP2B^Intron5^ mutant mouse model. When mutant CHMP2B expression was restricted to only neurons, 18-month-old mice showed a decreased density of synaptic vesicles, and the presence of enlarged endosomes, autophagosomes and electron-dense structures in presynaptic terminals by electron microscopy (EM). Additionally, by 24 months of age, synaptophysin distribution was altered in the NMJs of this mouse model, with synaptophysin staining noticeably more punctate in mutant CHMP2B NMJs, suggesting a decrease in the number of synaptic vesicles in the NMJ of the CHMP2B FTD model.^131^

Taken together, work using fly and mouse models of FTD-causative mutation in CHMP2B show that dysfunction in an endosomal trafficking protein can lead to deficits in presynaptic membrane trafficking.

### FTD/ALS

#### C9ORF72

The C9orf72 protein, encoded by the *C9ORF72* gene, is a Rab GTPase. Whilst its full function is not known, numerous cellular interactors have been demonstrated and it has been implicated in numerous intracellular trafficking functions including endocytosis, endosomal trafficking, lysosomal biogenesis and autophagy.^132-135^ These interactions reside in multiple intracellular locations, such as the nuclear membrane, cytoskeleton, mitochondria and membraneless organelles, and it plays key roles both in neurons and myeloid cells (reviewed in Smeyers *et al.*^136^).

An expanded GGGGCC intronic repeat mutation in the gene *C9ORF72* is the most common familial cause of both FTD and ALS.^12,13^ It has been proposed to mediate toxicity via three potential mechanisms: haploinsufficiency of *C9ORF72*, toxicity mediated via the production of repeat RNA, or production of toxic dipeptide repeat proteins (DPRs) via translation of repeat RNA.^137^ Despite intense scrutiny since the 2011 discovery of this mutation, no single mechanism has emerged as the clear driver of pathology. Instead, recent evidence suggests that a combination of these proposed mechanisms leads to disease. This ‘dual-hit’ model proposes that the loss-of-function of C9orf72 protein, which has multiple roles in endolysosomal/autophagy pathways, acts to increase vulnerability with toxic gain-of-function mechanisms.^136^

##### Synaptic pathophysiology in *C9ORF72*

Synaptopathy is established in *C9ORF72* mutation carriers and can be detected before the overt onset of disease-related symptoms. PET imaging with a radioligand, which binds to synaptic vesicle protein 2A (SV2A) showed reduced synaptic density in the thalamus in pre-symptomatic cases, with enhanced synaptic loss in the frontotemporal regions of a symptomatic individual.^138^

For the purpose of this review, we have summarized the evidence available in the literature for presynaptic synaptopathy in *C9ORF72* FTD/ALS and subdivided information into different potential disease mechanisms, with particular attention given to the nature of the disease model used in each study.

##### Loss of C9orf72 protein

The C9orf72 protein was first reported to have a presynaptic localization through observed co-localization with synaptophysin in human iPSC-derived motor neurons.^139^ Immunoprecipitation in this study also revealed interactions between C9orf72 and Rab3, a RAB that has a known function in the synaptic vesicle cycle,^140^ suggesting a role in presynaptic membrane trafficking for the C9orf72 protein.^139^

Modelling haploinsufficiency of the gene, an approximate 50% reduction of C9orf72 in zebrafish resulted in a significant reduction in synaptic vesicle protein 2 (SV2) at NMJs, with decreased synaptic vesicle turnover and reduced frequency of spontaneous neurotransmitter release.^141^ The authors additionally showed by immunoprecipitation that C9orf72 interacts with SV2A,^141^ a protein implicated in the sorting of synaptotagmin-1 to synaptic vesicles during endocytosis.^142,143^ In agreement with the previously identified interaction between C9orf72 and Rab3,^139^ C9orf72 depleted zebrafish show a reduction in the number and area of Rab3a positive puncta.

In addition to SV2A and Rab3, another synaptic vesicle-associated protein, synapsin, also interacts with C9orf72.^144^ Indeed, in the hippocampus of C9orf72 knockout mice, loss of synapsin was observed at 12 weeks of age. Ultrastructural analysis by EM in the CA3 region of mice revealed a significant decrease in synaptic vesicle density per synapse and a decrease in the number of docked vesicles.^144^ Furthermore, a decrease in the levels of C9orf72 and synapsin is observed in the hippocampus of patient tissue (C9orf72-ALS/FTD and FTD).^144^

Interestingly, a possible selective function of C9orf72 in excitatory synapses has been reported. A 50% reduction in C9orf72 protein in hippocampal neurons resulted in a significant decrease in the number of excitatory synapses with no effect on inhibitory synapses.^144^

##### Dipeptide repeat proteins

Five different DPRs are produced from unconventional repeat-associated non-ATG dependent (RAN) translation of sense and antisense *C9ORF72* repeat expansion RNA.^145-147^ This generates polypeptides of glycine-alanine (GA), glycine-proline (GP), glycine-arginine (GR), proline-arginine (PR) and alanine-proline (AP), which have different biophysical properties and toxicity profiles.^148^ One of the methods used to model the toxicity of the DPRs has been overexpression, with codon optimization used to allow the expression of individual DPRs. Using this approach, different lengths of GFP-tagged polyGA DPR were expressed in primary cortical and motor neurons, with live cell imaging revealing that shorter polypeptides are more mobile in neurites compared to longer ones.^149^ Additionally, polyGA aggregates were found to significantly impair the release of the synaptic vesicle dye FM4-64 despite increased Ca^2+^ influx, and displayed reduced SV2A while leaving the pre- and post-synaptic markers synaptophysin and PSD95 unaffected. Importantly, presynaptic phenotypes and toxicity were rescued by overexpression of the synaptic vesicle protein SV2A.^149^

##### Modelling multiple disease mechanisms

Patient-derived iPSC lines offer the advantage of the presence of the full human *C9ORF72* hexanucleotide repeat expansion and genomic context, and in conjunction with isogenic corrected controls (where the repeats have been excised), offer a powerful tool to more physiologically model all *C9ORF72* disease mechanisms.

When *C9ORF72* patient-derived iPSCs and isogenic corrected controls were differentiated into excitatory cortical neurons and co-cultured with primary mouse astrocytes, multi-electrode array analysis showed an increase in the rate of burst firing and altered network activity, with the size of the RRP found to be reduced in *C9ORF72* neurons.^150^ As mentioned above, C9orf72 interacts with synaptic vesicle trafficking proteins. Taken together, this suggests that loss of C9orf72 function may reduce neurotransmitter release. The authors additionally looked at synapses by quantifying the co-localization of synapsin and PSD-95, which showed an increase in synaptic densities in *C9ORF72* cortical neurons, resulting in elevated synaptic input.^150^ However, synapsin was recently described as an interactor of C9orf72,^144^ and thus alternative presynaptic markers should be tested to confirm global synapse alteration rather than accumulation of a C9orf72 protein interactor.

Catanese *et al.*^151^ performed transcriptomic analysis on human iPSC-derived motor neurons and found a reduction in the expression of synaptic genes and a time-dependent loss of excitatory synapses in *C9ORF72* mutant lines, highlighting that the age of cultures is important for interpreting synaptic pathophysiology.^151^ Indeed, *C9ORF72* iPSC derived motor neurons show hyperexcitability at 21 days *in vitro* (DIV) compared to controls, but this difference in firing rates is no longer significant at DIV 42, although they do still show a lower spike amplitude.^152^ In agreement with Catanese *et al.*, DIV 21 and DIV 42 *C9ORF72* motor neurons reveal a significant reduction in the expression of genes involved in neurotransmitter release and synaptic vesicle cycling (*RAB3B*, *SYT2*, *SYT7*, *SV2C* and *SYN3*).^152^

Further transcriptomic studies in post-mortem human tissue have revealed novel insights into disease mechanism. When comparing RNA-seq from post-mortem frontal cortex of FTD/ALS individuals, hits associated with vesicle transport were affected by the presence of *C9ORF72* mutation specifically.^153^ This finding is supported by a further study where nuclei without TDP-43 pathology in *C9ORF72* repeat expansion carriers revealed alterations to vesicle transport pathways.^154^ The connection from findings of dysfunction in global vesicular transport and synaptic vesicle pathways has not yet been determined, but these studies imply that *C9ORF72* mutation, through loss of C9orf72 protein or alternate pathways, causes downstream effects on vesicle trafficking pathways and are a significant disease relevant pathomechanism.

##### Non-cell autonomous factors

In addition to the abovementioned difficulties inherent in modelling the *C9ORF72* hexanucleotide repeat expansion, and the discussed importance of the maturity of the cultures used for iPSC-derived neuron studies, there are further confounding issues associated with the study of *C9ORF72*. Whilst it is outside the scope of this review to address these issues in detail, it is important to briefly note how these may impact our interpretation of *C9ORF72* presynaptic pathophysiology. In particular it is important to note that C9orf72, like progranulin, is highly expressed in microglia, and there is evidence to support that microglia can modulate synaptic pathophysiology.

Aged C9orf72 global knockout mice show cortical synaptic loss and learning and memory defects; however, in this system, contribution from neurons and glia cannot be dissected.^155^ In neuron-microglia co-cultures with C9orf72 selectively removed from microglia, enhanced synaptic pruning was observed, evidenced by an increase in the number of synaptophysin puncta within microglia.^155^ Additionally, in a microglia-specific C9orf72 knockout mouse, enhanced synaptic pruning was also observed in the motor cortex at 12 months old,^155^ indicating a key role for microglia.

Co-culture models, where control motor neurons plated with either *C9ORF72* patient, gene-corrected or control iPSC-derived astrocytes, have defined further cell autonomous roles for C9orf72.^156^ When control motor neurons were plated with *C9ORF72* mutant astrocytes, the authors observed a progressive loss of action potentials in motor neurons due to decreases in the magnitude of voltage-activated sodium and potassium currents, which was absent in co-cultures with gene-corrected astrocytes.^156^

Thus, the type of motor neuron culture, and possible contamination by astrocytes in culture, is another extremely important consideration for *C9ORF72* research. These important observations also highlight that non-neuron autonomous roles for C9orf72 in normal microglial and astrocyte function contribute to synaptic integrity in the ageing brain.

#### FUS

Mutations in fused in sarcoma (*FUS*) are found in 5% of ALS cases, which present with FUS pathology; conversely, FTD is not associated with *FUS* mutation but 10% of cases present with pathological FUS aggregates.^157-159^ The majority of FUS, a DNA/RNA binding protein, resides in the nucleus, where it plays roles in RNA metabolism^160^ and DNA damage repair.^161^ In addition to its nuclear role, FUS has multiple cytoplasmic roles,^162^ such as mRNA transport, microRNA processing and local translation.^40^ In neurons, FUS is implicated in the splicing of multiple synaptic RNAs.^163,164^ Dendritically localized FUS also regulates dendritic spine morphology by regulating plasticity related genes such as GluA1 and SynGAPα2.^165,166^

ALS causative mutations in FUS are largely found in the C-terminal nuclear localization signal (NLS) of the protein, a region that controls the nuclear-cytoplasmic shuttling of the FUS protein, and thus mutants often display cytoplasmic mislocalization prior to cytoplasmic aggregation.^167,168^

####  

##### Evidence that mutant FUS causes presynaptic aberrations comes from multiple model systems

In a *Caenorhabditis elegans* model overexpressing a C-terminal truncation variant of FUS (similar to that produced by ALS mutants), the ultrastructure of cholinergic motor neurons and GABA-ergic motor neurons were altered, with EM showing large endosome-like structures in the presynaptic terminal. Expression of human FUS (wild-type or mutant) was additionally found to modify the size, position and docking of synaptic vesicles at the NMJ.^169^ A zebrafish model for FUS ALS, where the *fus* orthologue is deleted, displayed impaired motor abilities, decreased motor neuron length and fragmentation of the NMJ,^170^ suggesting that loss of FUS can also affect presynaptic pathology. Interestingly, ALS patient-derived motor neurons with a FUS mutation showed increased synaptic accumulation of FUS compared to controls.^171^ Zebrafish larvae expressing the ALS-associated FUS mutation R521H were found to have impaired motor activity (swimming) and reduced quantal neurotransmitter release at the NMJ when compared to larvae expressing human wild-type FUS.^172^ Though confocal resolution did not detect any changes at the NMJ, super-resolution imaging using STED (stimulated emission depletion) microscopy found aberrant organization of the presynaptic protein Bruchpilot (the orthologue of the essential AZ protein ELKS/CAST/ERC protein) in R521C FUS expressing ALS mutant *Drosophila* larvae.^173^ Additional work with *Drosophila* showed that overexpression of either wild-type or ALS mutant FUS results in a decreased number of presynaptic active zones.^174^ However, up to this point there was no evidence for a direct role for FUS at the synapse.

The first study to show the FUS protein localizing to the presynaptic terminal in mammalian cells came in 2016 when STORM (stochastic optical reconstruction microscopy) super-resolution imaging verified its presence in cultured hippocampal neurons.^175^ This observation that FUS is physically present at the presynaptic terminal opened new possibilities for the fundamental role of FUS and the possible molecular mechanisms by which mutations in the protein lead to neurodegenerative disease. The presynaptic localization of FUS was corroborated in an independent study, verifying the role of FUS at neuron peripheral terminals at the NMJ.^176^ Interestingly, So *et al.*^176^ also looked for early presynaptic alterations in transgenic mice overexpressing human wild-type FUS and reported that presynaptic mitochondrial abnormalities are apparent by postnatal Day 6. By Day 15, these mitochondrial abnormalities are coupled with a loss of synaptic vesicles and synaptophysin protein.^176^ Loss of another presynaptic protein, synaptotagmin, was also seen in *Drosophila* neurons overexpressing mutant but not wild-type FUS.^177^ Conditional expression of wild-type or ALS-associated FUS variants in mouse models revealed that at postnatal Day 30, mutant NMJs showed significant presynaptic ultrastructural abnormalities, characterized by a significant reduction in the density of synaptic vesicles and a decrease in the number of morphologically normal mitochondria.^178^ Interestingly, a maturation-dependent alteration of FUS localization has been reported, which could account for progressive changes: using super-resolution imaging in rodent synapses, the localization of FUS was observed to be predominantly postsynaptic in the early stages of development, while in mature synapses, the protein was localized to the axonal terminal.^171^

*In vivo* studies on aged mouse models also corroborate a role for FUS at the synapse. EM analysis revealed defects in synapses at 22 months in heterozygous FUS NLS mutant mice, with motor cortex inhibitory and excitatory synapses showing a larger bouton size, smaller active zone and more synaptic vesicles per synapse in FUS mutant mice. Additionally, FUS accumulated in the synapse of mutant mice and displayed altered synaptosomal levels of some of its target transcripts.^179^ Further studies on the heterozygous FUS NLS mutant model showed increased synaptic FUS localization, with RNA-seq of the cortex and synaptoneurosomes revealing age-dependent alterations to synaptic mRNAs. Thus, this work showed a direct link between FUS and synaptic transcripts.^180^ These authors have optimzed a protocol for subcellular CLIP-seq to investigate subcellular-dependent changes in RNA bound to a target RNA binding protein, allowing the identification of the synaptic targets of FUS.^181^

Thus, numerous reports have now shown that FUS is localized to the presynaptic terminal,^171,175,182^ indicating a presynaptic physiological role for FUS. Additionally, a novel interaction for FUS with syntaphilin, an axonal mitochondrial tethering protein has been reported.^182^ Mitochondrial dysfunction is frequently reported in FTD/ALS, which highlights another pathway that could interact with synaptic pathophysiology.

#### TDP-43

Similar to FUS, mutations in TDP-43 are a rare cause of genetic ALS,^183^ but pathological aggregates of TDP-43 are the most frequently observed proteinopathy across FTD and ALS.^184^ TDP-43 is an RNA-binding protein predominantly found in the nucleus with broad roles in RNA processing, including roles in transcription, splicing, mRNA stability, transport and translation.^185^ TDP-43 regulates multiple mRNAs important for CNS development, synaptic plasticity and neurotransmission,^186^ and knockout of TDP-43 in mice is embryonic lethal.^187^ Nuclear depletion of TDP-43 concomitant with cytoplasmic aggregation is a hallmark of TDP-43 pathology.^18^ This loss of nuclear TDP-43 in conjunction with the appearance of cytoplasmic arguments means that TDP-43 FTD/ALS presents with elements of both loss- and gain-of-function.^188^

##### Evidence for synaptopathy in TDP-43 FTD/ALS comes from numerous model systems

The first evidence for synaptopathy in TDP-43 came from *Drosophila* lacking TAR DNA binding protein homologue (TBPH), the fly orthologue of TDP-43. Flies lacking TBPH presented with significantly reduced axonal branching and reduced synaptic boutons at the NMJ.^189^ To investigate the synaptic function of TBPH, Diaper *et al.*^190^ systematically compared loss- and gain-of-function of *Drosophila* TBPH. TBPH dysfunction caused impaired synaptic transmission at the larval NMJ and in the adult. Tissue-specific knockdown, together with electrophysiological recordings at the larval NMJ, also revealed that alterations of TBPH function predominantly affected presynaptic efficiency, suggesting that impaired presynaptic transmission is one of the earliest events in TDP-43-related pathogenesis.^190^ Reduced levels of the voltage-gated calcium channel, cacophony, mediate some of the physiological effects of TDP-43 loss in *Drosophila*, proposed to be due to altered splicing and increased degradation.^191^

Flies expressing human TDP-43 harbouring pathogenic mutations show increased HDAC6 expression, decreased Bruchpilot acetylation, larger vesicle-tethering sites and increased neurotransmission.^192^ As noted above, Bruchpilot is the orthologue of the essential mammalian active zone protein ELKS/CAST/ERC and forms a significant part of the synaptic density which tethers vesicles, and the deacetylation of Bruchpilot is controlled by HDAC6.^192^ Thus the effects mediated here may be reflective of secondary effects of TDP-43 pathology on HDAC6 mediating synaptic dysfunction.

Interestingly, overexpressing human TDP-43 mutants selectively in *Drosophila* motor neurons results in mutant TDP-43 sequestering Hsc70-4 mRNA and impairing its translation.^193^ Hsc70-4 has multiple roles in the synaptic vesicle cycle. In agreement, overexpression of wild-type or mutant TDP-43 results in reduced FM1-43 dye uptake at the larval NMJ, indicating defects in synaptic vesicle endocytosis.^193^ Overexpression of Hsc70-4, CSP (Hsc70-4’s co-chaperone) or Dynamin with the TDP-43 mutant were all able to rescue the defects in FM1-43 dye uptake suggesting upregulation of synaptic vesicle endocytosis is sufficient to rescue the phenotype.^193^

NeuroLNC, a neuron-specific long non-coding RNA, was identified in a *C. elegans* screen for long non-coding RNAs, which regulate synaptic vesicle release and synaptic activity. Interestingly the effects of neuroLNC on synaptic vesicle fusion require interaction with TDP-43.^194^

Expression of the ALS mutant TDP-43 G348C in zebrafish larvae led to impaired swimming and increased motor neuron vulnerability with reduced synaptic fidelity, reduced quantal transmission, and more orphaned presynaptic and postsynaptic structures at the NMJ.^172^ Interestingly, in this study, all behavioural and cellular features were stabilized by chronic treatment with L-type calcium channel agonists.^172^ Synaptic dysfunction also occurs in a zebrafish knockdown model of TDP-43, which showed altered quantal neurotransmitter release at the NMJ in larvae,^195^ suggesting these effects may be due to a loss of TDP-43 function.

Transgenic mice overexpressing mutant human TDP-43^A315T^ show behavioural deficits, which are associated with the accumulation of nuclear and cytosolic TDP-43 C-terminal fragments, a decrease in endogenous mouse TDP-43 levels, and synaptic loss.^196^ Transgenic mice over-expressing human wild-type neuronal TDP-43 also show reduced staining of the presynaptic protein synapsin 1 in the cortex. The authors additionally reported that expressing TDP-43 in SH-SY5Y cells results in reduced synapsin 1 expression by western blot, showing that this regulation occurs even in a non-neuronal context.^197^ Another mouse model, this time overexpressing TDP-43^Q331K^, showed early alterations in neurotransmission at the NMJ at 3 months and altered morphology of SV2 staining at the NMJ when compared to age-matched non-transgenic mice or transgenic mice expressing wild-type TDP-43.^198^

Elegant experiments utilizing an inducible transgenic mouse model expressing human TDP-43 lacking the NLS, which displays impaired nuclear import and thus mislocalization to the cytoplasm, support an axonal gain of function in TDP-43 ALS. When motor neurons from these TDP-43 NLS mutant mice were cultured in microfluidic chambers, the authors found that mislocalized TDP-43 co-localizes with Ras GTPase-activating protein-binding-protein1 (G3BP1) to form condensates along axons, and reported that this pathological mislocalization of TDP-43 disrupts axonal and synaptic protein synthesis.^199^ Unlike FUS, where numerous super-resolution imaging studies have shown it to be present at the presynaptic terminal, limited evidence exists showing TDP-43 is presynaptically localized, with only one paper showing TDP-43 immuno-EM presynaptic staining.^200^ Thus, its role in synaptic protein synthesis and synaptic vesicle function, may be indirect or via axonal disruption.

Clear and compelling evidence linking TDP-43 to synaptic physiological function in health and disease came from recent back-to-back publications, which showed that UNC13A cryptic exon splicing is controlled by TDP-43.^201,202^ UNC13A was initially identified as a genetic risk factor for ALS in 2009,^203^ and since then, multiple independent studies have reported that UNC13A is a modifier of survival in ALS.^204-206^ As discussed above, UNC13 is essential for synaptic vesicle fusion and neurotransmission,^207,208^ with its interaction with syntaxin required for synaptic transmission^209^ and other SNAREs for optimal SNARE complex assembly.^56-58^ Furthermore, the position of UNC13 within the active zone is important for both neurotransmitter release probability and kinetics.^210^ TDP-43 represses cryptic exon inclusion in UNC13A,^201^ and loss of nuclear TDP-43 leads to the inclusion of a cryptic exon in UNC13A.^202^ The inclusion of this cryptic exon leads to nonsense-mediated decay of the RNA transcript and a corresponding loss in UNC13A protein. The physiological effects of the loss of this synaptic vesicle-associated protein have yet to be described. However, this is a clear example of disrupted nuclear function resulting in impaired presynaptic synaptic vesicle functionality.

Together, TDP-43 modelling suggests that there may be both gain- and loss-of-function roles of TDP-43 mutation or pathology that result in presynaptic pathologies and synapse loss, predominantly by nuclear and axonal mechanisms. These mechanisms may have widespread impact due to the detailed high frequency of TDP-43 pathology in sporadic FTD/ALS. TDP-43 pathology also occurs in most genetic forms of FTD/ALS, with the exception of *MAPT*, *FUS* and *SOD1*, and thus in these cases could have additive effects on presynaptic pathology.

#### CHCHD10

Mutations in coiled-coil-helix-coiled-coil-helix domain containing 10 (CHCHD10), a nuclear encoded mitochondrial protein, are associated with both FTD and ALS.^211^ CHCHD10 is enriched in the cristae junctions,^211^ as part of the mitochondrial contact site and cristae organizing system complex.^212^ Initially reported as a genetic cause of ALS-FTD, further mutations in CHCHD10 have now been reported as causative for mitochondrial DNA instability disorder, late-onset spinal motor neuropathy and Charcot-Marie-Tooth disease type 2.^211^

When CHCHD10 was knocked down, or ALS-FTD-causative CHCHD10 variants (wild-type, R15L and S59L) expressed in primary hippocampal neurons, a reduction in synaptophysin staining was observed.^213^ Long-term synaptic plasticity deficits occur in CHCHD10^R15L^ and CHCHD10^S59L^ transgenic mice, also with decreased synaptophysin intensity staining observed in the CA3 region of the hippocampus.^214^ This work also reported interesting links between CHCHD10 and TDP-43; CHCHD10 mutation was found to drive TDP-43 pathology, with phospho-TDP-43 immunoreactivity significantly increased in CHCHD10 R15L and S59L mouse frontal cortex.^214^

It is unclear whether altered synaptic gene expression or protein levels are modulated via CHCHD10’s function in mitochondria. However, synaptic pathology may also be indirect via cytoplasmically mislocalized TDP-43, which has been reported in these models.^213^

### Amyotrophic lateral sclerosis

#### SOD1

Mutations in superoxide dismutase 1 (SOD1) account for up to 20% of genetic ALS cases, and to date ∼155 mutations have been identified.^215^ SOD1 is a metallo-enzyme that removes superoxide residues,^216^ and mutations lead to pathological aggregates of misfolded SOD1 in motor neurons and muscle^217^ Interestingly, SOD1 ALS does not present with TDP-43 pathology,^218-220^ suggesting an alternative pathogenesis in these cases. However, as outlined below, SOD1 models also present with presynaptic deficits, supporting a presynaptic convergence of pathomechanism at this intraneuronal location.

The most commonly used model of SOD1 ALS is the SOD1^G93A^ mouse. Studies have reported early alterations to the NMJ in this model. At a presymptomatic, stage when NMJs are still intact, abnormal mitochondria were seen in the NMJs of SOD1^G93A^ mice.^221,222^ By the point of NMJ denervation, the presynaptic terminal is reported to have fewer and larger mitochondria, and a reduction in the number of docked synaptic vesicles with no change to the overall number at the NMJ.^221,222^

Additional evidence is available from alternate models of SOD1 ALS. Ultrastructural analysis of NMJs in *C. elegans* transgenic for mutant human SOD1^G85R^ revealed a reduction in the number of synaptic vesicles.^223^ Further, SOD1^G85R^ expression in the giant squid synapse altered synaptic ultrastructure and inhibited synaptic transmission, reducing both the size of the RRP and mobility from the RP to the RRP of synaptic vesicles.^224^

Thus, SOD1 mutation can clearly alter synaptic vesicle and presynaptic functionality independent of TDP-43 accumulation, although the mechanisms of this are still unknown and warrant further investigation.

## Common pathomechanisms identifying the mechanistic link between ALS and FTD

**Figure 3:**
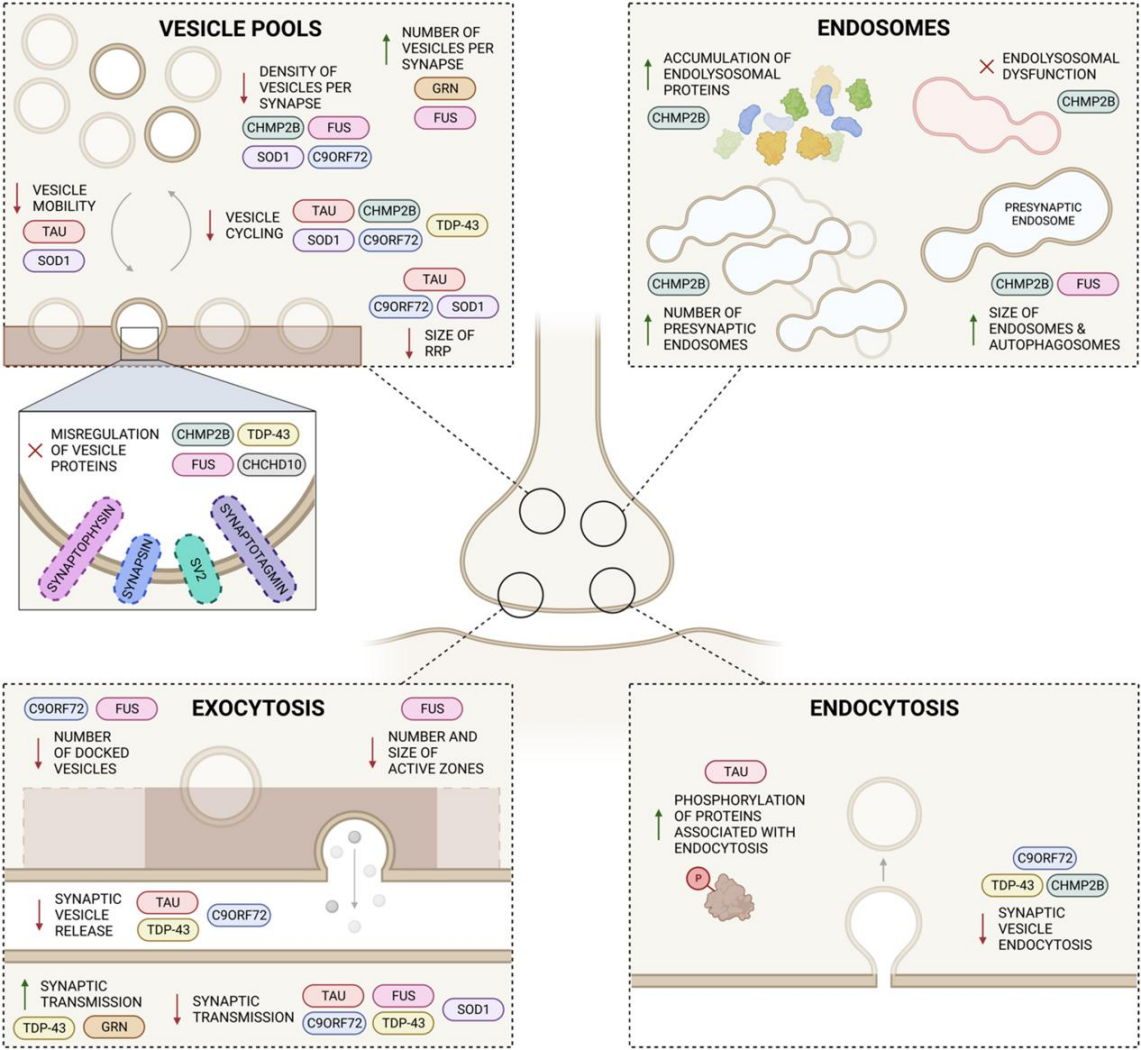
**Molecular mechanisms underlying presynaptic dysfunction in ALS and FTD pathogenesis**. Schematic representation of the presynaptic terminal showing the roles of amyotrophic lateral sclerosis (ALS)- and frontotemporal dementia (FTD)-associated genes in the synaptic vesicle cycle and the pathways that are dysregulated. Synaptopathy in ALS and FTD may be caused by misregulation of presynaptic vesicle pools, alterations to exocytosis and endocytosis, and dysregulation of the endolysosomal system, leading to disrupted synaptic activity and neuronal function. Created with BioRender.com.

ALS and FTD exist on a disease spectrum. At one spectrum end, ALS-associated genetic mutations are often grouped as disorders of RNA biology. At the other end, FTD-associated genetic mutations are often seen as disorders of the endolysosomal system. Exactly how these two diseases are linked at the level of molecular mechanisms is not fully understood. But it is abundantly clear from a genetic, pathological and familial perspective that these two diseases are complexly intertwined. Numerous publications in the field now clearly show that FTD/ALS disease-relevant dysfunction converges on the presynaptic terminal (depicted in Fig. 3). A recent comparison of the synaptic proteome from post-mortem spinal cord and human iPSC-derived motor neurons from patients carrying common mutations in ALS highlighted alterations to the synaptic vesicle trafficking machinery as a shared pathomechanism in ALS.^225^ The molecular mechanisms through which FTD and ALS causative mutations directly result in presynaptic defects remains to be fully defined, however, we have summarized the available evidence in this review.

Recent elegant advancements in our understanding of local protein translation have suggested a mechanistic link between FTD and ALS. Local protein translation is a ubiquitous feature of the presynaptic terminal,^226^ and the maintenance of homeostasis via efficient local protein translation and degradation is essential for synaptic health.^118^ Cioni *et al.*^227^ showed that the endolysosome is a site of local protein translation and Liao *et al.*^228^ demonstrated RNA granules hitchhiking on the endolysosome, allowing the transport of RNA to distal axonal locations. Crucially, the latter paper also identified the protein that connects RNA granules to the endolysosome as Annexin A11.^228^ Incredibly, Annexin A11 is a genetic cause of ALS, FTD and multisystem proteinopathy.^229-231^ The identification of Annexin A11 as the protein that attaches RNA granules to the endolysosome for distal transport in neurons posits this protein as both the physical and the metaphorical link between ALS and FTD, directly linking RNA biology and endolysosomal dysfunction. Although dysfunction of this process could thus be proposed to impact axo-synaptic function and specifically the localized signalling pathways at the presynaptic terminal, this remains to be directly investigated. Emerging evidence, however, strongly indicates that axo-synaptic translation is critical for neuronal maintenance and survival, and that FTD and ALS causative mutations impact mRNA transport, localization and local protein translation, which is necessary for maintenance of axons and synapses.^232^

Another important point of convergence may be through alterations to the biophysical properties of proteins. A key example of this is liquid-liquid phase separation (LLPS) behaviour. LLPS is the process by which multivalent interactions between soluble proteins (and frequently nucleic acids) allow their condensation into a separate phase from the rest of the dilute solute. This feature enables the formation of membraneless organelles, such as the nucleolus, the nuclear pore and RNA granules—all known to be affected in FTD/ALS.^233^ Indeed, many proteins associated with FTD and ALS contain intrinsically disordered domains, which permit multivalent interactions and LLPS. Further, disease-associated mutations often impair LLPS and the dynamics of membraneless organelles.^234,235^ Disrupted LLPS and reduced stress granule dynamics of FUS and TDP-43 have been associated with their fibrillization and aggregation,^236-239^ although it is unclear whether this occurs directly at stress granules or as an indirect consequence or being excluded from impaired stress granules.^240-242^ The *C9ORF72* repeat expansion associated DPRs can also impact LLPS and alter multiple membraneless organelles and their function.^235,243^ Aside from the classic RNA binding protein-associated LLPS, tau also undergoes LLPS, which may play a role in polymerization into microtubule bundles, but also in tau aggregation in disease.^244,245^ LLPS of SOD1 may also underlie its aggregation,^246^ and granulins, proteolytic products of the progranulin protein, may modify TDP-43 aggregation.^247^ It is important to note that LLPS can be tuned by environmental conditions (pH, ionic, ATP etc.) and post-translational modifications,^248^ which are all frequently altered in disease and thus may contribute to pathogenesis. Finally, as RNA transport granules are membraneless organelles, disrupted LLPS may also contribute to their impaired transport and association with local translation and, thus, axo-synaptic pathologies.^249^

Although it is not yet clear how LLPS contributes to synaptopathy, recent evidence shows that at the presynaptic terminal, tau undergoes activity-dependent LLPS to form nanoclusters that selectively control the mobility of recycling vesicles.^250^ As multiple other LLPS-associated proteins implicated in FTD and ALS are also found at the presynaptic terminal, the impact of these nano-biomolecular condensates on the physiology and pathophysiology of the presynaptic terminal warrants further investigation.

## Perspective

There is now a significant body of evidence showing that presynaptic dysfunction is prominent in multiple genetic models and post-mortem tissue in FTD and ALS. Figure 3 illustrates intersections where presynaptic dysfunction has been identified across model systems. Crucially, the physiological role of many of these ALS- and FTD-associated proteins at the presynaptic terminal is not well understood. What is clear is that synapse loss is an early event in ALS and FTD and occurs before the onset of symptoms. As collated here, presynaptic dysfunction is a common pathomechanism across multiple genetic forms of disease. This highlights that a better understanding of the fundamental physiological roles of proteins, including at the presynapse, is needed to better define the pathophysiological consequences of genetic mutation and synaptopathy. Enhanced understanding will aid efforts to identify novel therapeutic targets with the potential to impact early disease pathways, which intersect at the presynaptic terminal and prevent or delay the loss of neuronal connectivity and clinical symptoms in FTD and ALS, and more broadly across neurological disorders.
